# Serological Evidence of Selected Tick-Borne Pathogens and *Dirofilaria immitis* in Owned Dogs from Italy and Greece

**DOI:** 10.3390/vetsci13020133

**Published:** 2026-01-29

**Authors:** Angela Di Cesare, Chiara Astuti, Simone Morelli, Donato Traversa, Antonio Frangipane di Regalbono, Giulia Simonato, Donatella Damiani, Ilaria Lallone, Anastasia Diakou

**Affiliations:** 1 Department of Veterinary Medicine, University of Teramo, 64100 Teramo, Italy; castuti@unite.it (C.A.); smorelli@unite.it (S.M.); dtraversa@unite.it (D.T.); ddamiani1@unite.it (D.D.); ilallone@unite.it (I.L.); 2 Department of Animal Medicine, Production and Health, University of Padua, 35020 Padua, Italy; antonio.frangipane@unipd.it (A.F.d.R.); giulia.simonato@unipd.it (G.S.); 3 School of Veterinary Medicine, Faculty of Health Sciences, Aristotle University of Thessaloniki, 54124 Thessaloniki, Greece; diakou@vet.auth.gr

**Keywords:** epidemiology, CVBDs, dogs, Italy, Greece

## Abstract

Canine vector-borne diseases (CVBDs) are caused by pathogens transmitted by several invertebrates, posing a significant threat to both animal and human health worldwide. In recent years, the geographical distribution of CVBDs has changed in many countries, driven by climate change, increased pet travel, movements of goods, and anthropization of wildlife habitats. This study investigated the exposure to major CVBDs in 423 owned dogs from Italy and Greece. Individual serum samples were analyzed using serological methods. The SNAP^®^ 4Dx IDEXX test was used to detect *Dirofilaria immitis* circulating antigens and antibodies against *Anaplasma* spp., *Ehrlichia* spp. and *Borrelia burgdorferi*. Additionally, an indirect immunofluorescence antibody test (IFAT) was used to detect antibodies against *Rickettsia conorii* and *Babesia canis*. Overall, 171 (40.4%) dogs were positive for at least one pathogen. Antibodies against *R. conorii*, *Ehrlichia* spp., *Anaplasma* spp., *B. canis* and *B. burgdorferi* were detected in 118 (27.9%), 28 (6.6%), 29 (6.8%), 5 (1.2%) and 3 (0.7%) dogs, respectively. *Dirofilaria immitis* antigens were found in 7 dogs (1.6%). A Binomial Logistic Regression was performed and revealed a statistically significant association between age (dogs > 7 years old) (*p* = 0.005; OR = 1.903; 95% CI = 1.215–2.2981) and presence of at least one clinical sign (*p* = 0.028; OR = 4.082; 95% CI = 1.168–14.262) and positivity to at least one vector-borne pathogen. These findings confirm that dogs in both Italy and Greece are exposed to a range of vector-borne pathogens and highlight the importance of continuous epidemiological surveillance in European regions.

## Simple Summary

Canine vector-borne diseases (CVBDs) represent a threat to both animal and human health. The geographical distribution of these diseases has recently changed and expanded in many countries due to multiple driving factors. The present study aimed to investigate the seroprevalence of selected CVBDs in owned dogs from Italy and Greece. The results of the study confirm that dogs living in these countries are exposed to several vector-borne pathogens. Therefore, continuous epidemiological surveillance is essential to generate updated data and support the implementation of appropriate control strategies.

## 1. Introduction

Canine vector-borne diseases (CVBDs) transmitted by blood-feeding arthropods are an increasing concern in veterinary medicine and public health. They are caused by various pathogens, including parasites, bacteria, and viruses [1]. In Europe, dogs are exposed to vector-borne parasites, including the nematode *Dirofilaria immitis* and the protozoan *Babesia* spp., which are transmitted by mosquitoes and ticks, respectively. Several tick-borne bacteria are also widespread in Europe, including *Ehrlichia* spp., *Anaplasma* spp., *Borrelia* spp. and *Rickettsia* spp. [1,2,3,4]. In recent years, the distribution of CVBDs has changed considerably, with cases now being reported in areas previously considered non-endemic. This expansion is largely influenced by climate change, which increases the ecological suitability of new areas for arthropod vectors. Changing climatic conditions also extend the seasonal activity of vectors, allowing them to remain active beyond the warmer months [1,5,6]. Additionally, the increasing frequency of pet travel with their owners has facilitated the introduction of pathogens into new areas, especially when dogs are not adequately protected by preventive or therapeutic measures. Similarly, globalization has significantly contributed to this phenomenon through the increased movement of people, animals, and goods at an international level [4,7]. Another factor that has played a crucial role is the urbanization of natural habitats, which has significantly increased the likelihood of interactions among domestic animals, wildlife, and arthropod vectors [7,8].

Vector-borne pathogens (VBP) pose a serious threat to both animal and human health. Infected dogs may exhibit a broad spectrum of clinical manifestations, ranging from subclinical to severe, potentially life-threatening disease [9]. The clinical course may be exacerbated by co-infections. These conditions, resulting from simultaneous exposure to multiple infected arthropods or from the transmission of different pathogens by a single species, can complicate diagnosis, lead to more severe clinical signs, and negatively affect treatment efficacy [10]. Subclinically infected dogs may act as reservoirs, thereby contributing to the spread of CVBDs in both enzootic and free regions [9]. Moreover, many of these pathogens also have zoonotic potential and can cause severe disease in humans, including Lyme disease, Mediterranean Spotted Fever, and human granulocytic anaplasmosis, caused by *Borrelia burgdorferi*, *Rickettsia conorii*, and *Anaplasma phagocytophilum*, respectively [2,11,12].

Despite the growing relevance of CVBDs, epidemiological data remain limited or outdated in some European regions and require continuous updates. Regular serological surveillance in dogs represents a valuable tool for understanding the circulation of major CVBDs in specific areas, as dogs act as effective epidemiological sentinels [8,9]. In this context, recent studies have also documented the circulation of several VBP and hematophagous vectors in wild canids in Italy and Greece, further supporting the endemic circulation of these agents in the Mediterranean area [13,14]. This is particularly relevant in Italy and Greece, where climatic conditions are highly favorable to vector survival and pet and human movement related to tourism is intense [15].

Although numerous studies have investigated the epidemiology of CVBDs in different regions of Italy, the currently available data require updating. Information on these pathogens is indeed scarce in specific geographic areas [4,16,17,18]. Similarly, in Greece, epidemiological data on these diseases remain limited and require updating. Particular attention should be given to the northern regions of the country, which are characterized by environmental conditions favorable to vector development [19,20,21]. In this context, the present study investigated the seroprevalence of selected CVBDs in owned dogs from Italy and Greece in order to provide updated epidemiological data in at-risk canine populations.

## 2. Materials and Methods

**Figure 1:**
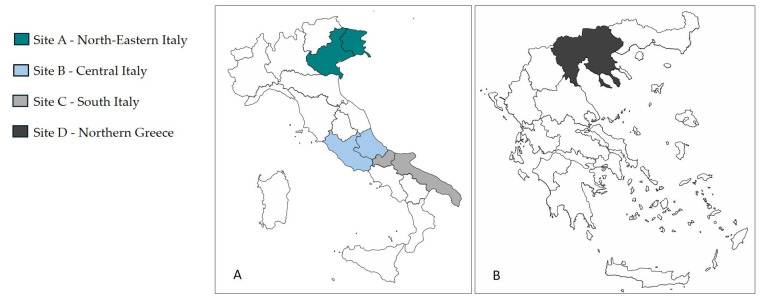
Study areas included in the present study: Italy (**A**) and Greece (**B**).

Between August 2022 and November 2023, a total of 423 owned dogs from Italy and Greece were enrolled in the study. Specifically, 89 dogs from northeastern Italy (Friuli Venezia-Giulia and Veneto regions, Site A), 145 dogs from central Italy (Abruzzo and Latium regions, Site B), 119 dogs from southern Italy (Molise and Apulia regions, Site C) and 70 dogs from regions of northern Greece (Site D) (Figure 1).

For each dog, data on age, sex, breed, access to the outdoor environment, and preventive ectoparasitic treatments received in the year preceding the sampling were registered. A physical examination was performed, and any clinical signs suggestive of vector-borne diseases were recorded. Dogs were enrolled during routine medical examinations conducted by local veterinarians, and all dog owners signed a written consent form before sampling. The inclusion in the study was random.

Individual blood samples were collected from each animal by venipuncture of the cephalic vein and transferred into tubes without anticoagulant. After clot formation, the samples were centrifuged for serum separation. The sera were stored at 2–4 ℃ until examination, performed no later than 3 days after collection [22]. For all the serum samples, the SNAP^®^ 4Dx (IDEXX Laboratories, Inc., Westbrook, ME, USA) was used to detect *D. immitis* circulating antigen and antibodies against *A. phagocytophilum*/*Anaplasma platys*, *Ehrlichia canis*/*Ehrlichia ewingii*, and *B. burgdorferi*, and the Indirect Immunofluorescence Antibody Test (IFAT) was used to detect antibodies (IgG) against *Babesia canis* (MegaFLUO^®^ BABESIA canis-Megacor Diagnostik GmbH, Hörbranz, Austria) and *R. conorii* (MegaFLUO^®^ RICKETTSIA conorii-Megacor Diagnostik GmbH, Hörbranz, Austria). The screening dilutions were set at 1:160 for both IFATs, according to the manufacturer’s instructions.

A Binomial Logistic Regression analysis was performed using Jamovi (Version 2.5—“the Jamovi project”, Sidney, Australia, 2024) to evaluate potential statistically significant associations between positivity to at least one vector-borne pathogen and selected potential risk factors (i.e., presence of clinical signs, prevention against ectoparasites, age, sex). The strength of associations was measured using the Odds Ratio, and a 95% confidence interval (CI) was calculated. Associations were considered significant when *p* ≤ 0.05.

## 3. Results

The dogs examined comprised 207 (48.9%) males and 216 (51.1%) females, with a mean age of 7 years. Specifically, 243 dogs (57.4%) aged 7 years or older, while 180 dogs (42.6%) were younger than 7 years. Three hundred and twenty-eight (77.5%) dogs were purebred, while ninety-five (22.5%) were mixed-breed. All dogs had regular access to the outdoor environment. One hundred and ninety-four (45.9%) dogs underwent antiparasitic treatments effective against ectoparasites in the year preceding the sampling.

A total of 171 dogs (40.4%) tested positive for at least one pathogen. Antibodies against *R. conorii* were found in 118 (27.9%) dogs, while 29 (6.8%), 28 (6.6%), 7 (1.6%), 5 (1.2%) and 3 (0.7%) dogs were positive for *Anaplasma* spp., *Ehrlichia* spp., *D. immitis*, *Babesia* spp. and *B. burgdorferi*, respectively (Table 1). Specifically, 155 (36.6%) were seropositive for a single pathogen, while 16 (3.8%) dogs tested positive for two or more VBPs (Table 2). Specifically, 5 dogs (1.2%) were positive for *Anaplasma* spp. and *Ehrlichia* spp. (1 from site A, 2 from site B, and 2 from site C), 3 (0.7%) were positive for *Anaplasma* spp. and *R. conorii* (1 from site A and 2 from site B), 5 (1.2%) for *Ehrlichia* spp. and *R. conorii* (all from site C), and 3 (0.7%) for *Anaplasma* spp., *Ehrlichia* spp., and *R. conorii* (i.e., 1 from site B and 2 from site C). Detailed results on dogs seropositive for more than one pathogen are shown in Table 2.

At least one clinical sign was observed in 36 dogs (8.5%), and 23 dogs (5.4%) showed two or more clinical signs. The most frequently reported signs were lethargy (52.8%), fever (27.8%), and weight loss (27.8%). Moreover, of the 171 dogs that tested positive for at least one pathogen, 23 (5.4%) showed clinical signs (Table 3).

Of the 194 dogs receiving preventive ectoparasitic treatments, 80 (41.2%) tested positive for at least one pathogen.

The Binomial Logistic Regression revealed a statistically significant association between age (i.e., dogs > 7 years old) (*p* = 0.005; OR = 1.903; 95% CI = 1.215–2.2981) and presence of at least one clinical sign (*p* = 0.028; OR = 4.082; 95% CI = 1.168–14.262) and positivity to at least one vector-borne pathogen. The results of the statistical analysis are detailed in Table 4.

## 4. Discussion

The results of this study confirm that owned dogs living in Italy and northern Greece are exposed to a range of VBPs, highlighting the ongoing circulation of these infections in Mediterranean areas. Based on previous epidemiological studies conducted in Italy and Greece and given the endemicity of canine vector-borne diseases in the Mediterranean area, exposure to several of the investigated pathogens was here. Accordingly, the present findings should be interpreted in relation to local epidemiological conditions in the investigated areas rather than prevalence at the national level [1,6,23].

Among the investigated pathogens, *R. conorii* was the most frequently detected in both Italy and Greece. The high seropositivity detected in both countries is consistent with the values found in previous studies during the last decade [3,4,12,24,25] and reflects the geographical distribution of its primary vector, *Rhipicephalus sanguineus* s.l., which is widespread throughout the Mediterranean basin and active year-round [1,25,26]. Although canine infection with *R. conorii* is most often subclinical, this pathogen is of high zoonotic importance, as it is the etiological agent of Mediterranean Spotted Fever, a disease that may cause severe clinical conditions in humans. Therefore, serological monitoring in dogs represents a valuable tool for assessing the geographical distribution of *R. conorii* and estimating the potential risk of human exposure [25,27,28]. The high seropositivity observed for *R. conorii* in both countries was expected and is consistent with data reported from other Mediterranean regions characterized by widespread circulation of *R. sanguineus* [23,27]. Although the here observed seroprevalence likely reflects a high level of exposure to *R. conorii*, serological cross-reactions with other *Rickettsia* species, e.g., *Rickettsia felis* or *Rickettsia typhi* may lead to false positive results. Therefore, it cannot be excluded that the seropositivity rates obtained here for *R. conorii* may have been overestimated [4,12].

No dogs from Italy tested positive for *B. canis* in this study, in contrast to previous reports over the past decade documenting high seroprevalence throughout the country [4,25,29,30,31,32]. The absence of seropositive dogs in the present study may be related to the exclusive inclusion of owned dogs. Indeed, dogs coming from shelters and kennels are at higher risk of VBPs, likely due to their lifestyle, the high animal density and the inadequate administration of antiparasitic treatments [4,29,31]. However, previous reports of higher seroprevalence in dogs from central and southern Italy do not reflect the distribution of the pathogen’s primary vector, the tick *Dermacentor reticulatus*. This tick species is predominantly distributed in northern Italy, whereas its presence in central and southern regions remains poorly documented. Therefore, the high seropositivity rates previously observed in central and southern Italy might be attributed to serological cross-reactions with *Babesia vogeli*, which is more commonly reported in these areas and is transmitted by *R. sanguineus* [32,33,34]. A similar scenario may also apply to Greece. Although antibodies against *B. canis* were detected in this study in Greece, the absence of *D. reticulatus* in the country, along with the known circulation of *B. vogeli* and *R. sanguineus,* suggests that cross-reactions may have contributed to the observed seropositivity [3,21]. Accordingly, the real distribution of *B. canis* remains unclear, and further surveys using species-specific tools are warranted to clarify the true circulation of this pathogen, along with studies aiming to investigate the potential role of other tick species in the transmission of *B. canis*. Even though the latter is known to occur within the study areas, the simultaneous presence of other species that may produce serological cross-reactions should be taken into account, and the results should be interpreted cautiously. For instance, cross-reactions between *B. canis* and *B. vogeli* have been described in other regions of the Mediterranean basin where *B. vogeli* is more widely distributed; on the contrary, *B. canis* and its vector are predominantly found in central and eastern Europe [23,33].

The seroreactivity to *Ehrlichia* spp. detected in the present study likely reflects exposure to *E. canis*, which is the main *Ehrlichia* species infecting dogs in Europe [9]. Specifically, the observed seroprevalence of *E. canis* is consistent with previous findings from the past decade showing no occurrence in northern Italy and high prevalence rates in southern and central regions of Italy [4,34,35]. The high positivity values for *E. canis* observed in Greece in the present survey agree with the seropositivity rates reported in previous investigations [20,36]. However, previous studies carried out in southern Italy reported a higher prevalence rate than that here observed. This finding may be partially explained by differences in the sampled population, as the present study mainly focused on owned dogs, whereas previous investigations included a high proportion of sheltered dogs, which are at increased risk of infection [10,24,29]. As for *R. conorii*, the seropositivity rates for *E. canis* detected in the present study in both Italy and Greece are consistent with those reported in other areas of the Mediterranean basin, where its vector is widely distributed [2,23].

Antibodies against *Anaplasma* spp. were detected with variable prevalence across the investigated areas of Italy and Greece, in agreement with previous reports published during the last ten years [3,15,24,34]. The main vector of *A. platys*, i.e., *R. sanguineus*, is distributed throughout Italy and Greece, while *Ixodes ricinus*, the vector of *A. phagocytophilum*, occurs mainly in northern regions in Italy and with relatively low populations in northern Greece [2,15,26,37]. The prevalence values here observed for this pathogen were expected in both investigated countries. Overall, *Anaplasma* spp. are widely distributed across countries of the Mediterranean basin, and the positivity rates found here are in line with previous data [2,23].

As emerged in previous studies conducted over the past decade, *B. burgdorferi* was here detected at low positivity rates throughout the Italian peninsula [11,38]. However, previous studies have reported a higher seroprevalence rate in northern Italy [4,25]. The higher rates are consistent with the geographical distribution of its vector, *I. ricinus*, which is more widespread in northern Italy. These data are of particular public health relevance, as this pathogen is the causative agent of human Lyme disease [25,26]. In Greece, *B. burgdorferi* was not detected in any of the enrolled dogs, a result that is consistent with previous surveys conducted [3,9,15]. This result may be associated with the limited presence of *I. ricinus* in the country [15,20]. Overall, the results for *B. burgdorferi* observed in the present study agree with data from other Mediterranean countries, where this pathogen is rarely detected [6,23].

The detection of *D. immitis* exclusively in central Italy, consistent with the recently reported southward expansion of the geographical range of the parasite, and at a low prevalence, contrasts with findings from other studies [4,34,35]. Nevertheless, the absence of positive dogs in other areas may be explained by the fact that all dogs included in the present study were privately owned and therefore more likely to receive regular preventive treatment [9,39,40]. A recent study has described the first case of macrocyclic lactone-resistant *D. immitis* in a dog in Europe, a phenomenon previously reported in the southern USA [41]. Therefore, regardless of the level of infection in various territories and the categories of dogs living in endemic areas, constant epidemiological surveillance for canine heartworm is crucial. Canine cardiopulmonary dirofilariosis is endemic in Greece, with prevalence in the hyperendemic, northern regions of the country ranging from 6.1% to 68%, in line with the prevalence value observed in the present study [9,19,42]. Similarly, to the trend observed in Italy, the geographic distribution of *D. immitis* is expanding southward in regions where its prevalence was very low until a few years ago [43]. Based on data reported over the past decade on the distribution of *D. immitis* in Italy, a higher prevalence of this nematode was expected in the central and southern regions of the country than the one observed in the present study. The absence of positive dogs in northern Italy is likely attributable to strict preventative measures adopted over time in this well-known endemic area. In contrast, chemoprophylaxis against canine cardiopulmonary dirofilariosis is not yet routinely performed in central and southern Italy [39,40]. Conversely, the prevalence observed in Greece was expected, given the endemicity of the parasite in the country [9,19,42]. In the Mediterranean basin, which is considered an endemic area for *D. immitis*, variable positivity rates for the parasite were reported. Moreover, over the past decade, canine cardiopulmonary dirofilariosis has progressively spread to eastern and northeastern European countries, some of which are now considered endemic [44,45].

A statistically significant association was observed between seropositivity to at least one pathogen and the age of dogs. This finding is most likely attributable to the longer period of exposure of older dogs to vectors and the pathogens they transmit, compared to younger dogs [46]. Even though VBPs often induce non-specific clinical signs in dogs [18], a statistically significant association was found between the presence of at least one clinical sign observed in the dogs investigated in the present study and seropositivity to one or more vector-borne pathogens. This finding indicates that the presence of even only one sign among those investigated should be taken into account when considering the possible involvement of VDBs and the assessment of exposure in dogs presenting nonspecific clinical signs, particularly in endemic settings. As commonly reported in the literature, co-infections were detected in this study. Co-infections may hinder accurate diagnosis in clinically ill dogs, as VBDs can cause overlapping clinical signs [10,18]. Specifically, co-infections involving *Ehrlichia* spp. and *Anaplasma* spp., as well as *Ehrlichia* spp. and *R. conorii*, were the most frequently detected, likely reflecting the shared transmission by the same arthropod vectors. Moreover, co-positivity for all three pathogens was observed in some dogs [2,37].

Although many dog owners in this study reported using antiparasitic formulations containing ectoparasiticidal compounds, a significant proportion of dogs tested positive for at least one pathogen. Given the lack of detailed information on preventive protocols applied by the owners, seropositivity to VBDs may be associated with inadequate or irregular administration of ectoparasiticides and repellents. The correct application of ectoparasiticides and repellents represents the most effective strategy to minimize the risk of diseases transmitted by arthropod vectors [4]. Moreover, periodic monitoring of dogs, even in the absence of clinical signs, represents an important control strategy. Greater attention should be directed to categories of animals at higher risk of VBDs infection, such as hunting dogs and those from kennels and shelters [4,9,31].

Several diagnostic tools are available for the detection of CVBP, with sensitivity and specificity varying depending on the target pathogen and the stage of infection. Therefore, comparisons between epidemiological studies should be interpreted with caution, as differences in diagnostic techniques, the geographical area investigated, and the characteristics and size of the sampled population may lead to over- or underestimation of the reported positivity rates. As demonstrated in previous surveys, the combined use of multiple diagnostic approaches, including molecular methods, is recommended to improve both diagnostic accuracy and to obtain more reliable epidemiological data [35].

In conclusion, the present study provides updated epidemiological data on major VBDs in dogs from Italy and Greece. Given the continuously evolving epidemiological scenario and the potential impact of these infections on animal and human health, ongoing surveillance is essential to identify and monitor areas at risk of infection [24]. Although some pathogens, such as *B. canis*, *B. burgdorferi*, *Anaplasma* spp., and *D. immitis*, were detected with low positivity rates, their clinical and zoonotic importance warrants continuous epidemiological attention [11,33,34]. Moreover, monitoring the distribution of arthropod vectors and their vector competence, along with increasing awareness among veterinarians and health authorities is essential for implementing effective surveillance strategies and safeguarding both human and animal health within a One Health perspective [21]. Further studies are needed to standardize diagnostic methods and to generate updated epidemiological data on CVBDs, particularly in regions where information remains limited.

## Figures and Tables

**Table 1:** Results of serological examinations (SNAP^®^ 4Dx rapid test and Indirect Immunofluorescence Antibody Test, IFAT): number (n), percentage (%) and 95% confidence intervals (CI) of dogs seropositive for *Dirofilaria immitis*, *Anaplasma phagocytophilum*/*Anaplasma platys*, *Ehrlichia canis*/*Ehrlichia ewingii*, *Borrelia burgdorferi*, *Rickettsia conorii* and *Babesia canis*.

	SNAP 4DX				IFAT			
Site	*An* n/tot (%; CI)	*Eh* n/tot (%; CI)	*Bb* n/tot (%; CI)	*Di* n/tot (%; CI)	*Rc* n/tot (%; CI)	*Bc* n/tot (%; CI)	Mixed VBP n/tot (%; CI)	Total VBP n/tot (%; CI)
**A**	8/89 (9.0; 0.0396–0.1695)	- -	1/89 (1.1; 0.0003–0.0610)	- -	27/89 (30.3; 0.2103–0.4099)	- -	1/89 (1.1; 0.0003–0.0610)	35/89 (39.3; 0.2913–0.5025)
**B**	11/145 (7.6; 0.0385–0.1317)	7/145 (4.8; 0.0196–0.0969)	1/145 (0.7; 0.0002–0.0378)	1/145 (0.7; 0.0002–0.0378)	28/145 (19.3; 0.1323–0.2669)	- -	4/145 (2.7; 0.0076–0.0691)	43/145 (29.6; 0.2236–0.3780)
**C**	8/119 (6.7; 0.0295–0.1282)	13/119 (10.9; 0.0595–0.1796)	1/119 (0.8; 0.0002–0.0459)	- -	51/119 (42.8; 0.3383–0.5225)	- -	9/119 (7.6; 0.0352–0.1387)	62/119 (52.1; 0.4275–0.6134)
**D**	2/70 (2.8; 0.0035–0.0994)	8/70 (11.4; 0.0507–0.2128)	- -	6/70 (8.6; 0.0321–0.1773)	12/70 (17.1; 0.0918–0.2803)	5/70 (7.1; 0.0236–0.1589)	2/70 (2.8; 0.0035–0.0994)	31/70 (44.3; 0.3241–0.5666)
**Total**	29/423 (6.8; 0.0464–0.0970)	28/423 (6.6; 0.0444–0.0943)	3/423 (0.7; 0.0015–0.0206)	7/423 (1.6; 0.0067–0.0338)	118/423 (27.9; 0.2255–0.3120)	5/423 (1.2; 0.0038–0.0274)	16/423 (3.8; 0.0218–0.0607)	171/423 (40.4; 0.3571–0.4527)

*An*: *Anaplasma* spp.; *Eh*: *Ehrlichia* spp.; *Bb*: *Borrelia burgdorferi*; *Di*: *Dirofilaria immitis*; *Rc*: *Rickettsia conorii*; *Bc*: *Babesia canis*.

**Table 2:** Number (n) and percentage (%) of dogs seropositive for two or more vector-borne pathogens (VBPs) in the present study.

Multiple Exposure	n (%)
*Anaplasma* spp. + *Ehrlichia* spp.	5 (1.2)
*Anaplasma* spp. + *Rickettsia conorii*	3 (0.7)
*Ehrlichia* spp. + *Rickettsia conorii*	5 (1.2)
*Anaplasma* spp. + *Ehrlichia* spp. + *Rickettsia conorii*	3 (0.7)

**Table 3:** Number (n) and percentage (%) of dogs presenting clinical signs and their corresponding positivity to vector-borne pathogens (VBPs).

Clinical Signs	N. of Dogs n/tot (%)	*An* n/tot (%)	*Eh* n/tot (%)	*Bb* n/tot (%)	*Di* n/tot (%)	*Rc* n/tot (%)	*Bc* n/tot (%)	Negative n/tot (%)
Fever	10/423 (2.4)	2/29 (6.9)	1/28 (3.6)	-	-	3/118 (2.5)	-	4/72 (5.5%)
Weight loss	10/423 (2.4)	3/29 (10.4)	-	-	-	2/118 (1.7)	-	5/72 (6.9%)
Splenomegaly	3/423 (0.7)	1/29 (3.4)	-	-	-	1/118 (0.8)	-	2/72 (2.8)
Ocular signs	3/423 (0.7)	-	-	-	-	1/118 (0.8)	-	2/72 (2.8)
Anorexia	5/423 (1.2)	1/29 (3.4)	-	-	-	2/118 (1.7)	-	2/72 (2.8)
Lameness	6/423 (1.4)	2/29 (6.9)	-	-	-	3/118 (2.5)	-	1/72 (1.4)
Hepatomegaly	1/423 (0.2)	1/29 (3.4)	-	-	-	1/118 (0.8)	-	-
Lethargy	19/423 (4.5)	7/29 (24.1)	2/28 (7.1)	-	-	6/118 (5.1)	-	5/72 (6.9)
Vomiting/diarrhea	1/423 (0.2)	-	-	-	-	1/118 (0.8)	-	-
Pale mucous membranes	6/423 (1.4)	1/29 (3.4)	1/28 (3.6)	-	-	1/118 (0.8)	-	3/72 (4.2)
Lymphadenomegaly	8/423 (1.9)	5/29 (17.2)	1/28 (3.6)	-	-	-	-	2 (2.8)
Skin lesions	1/423 (0.2)	-	-	-	-	-	-	1 (1.4)

*An*: *Anaplasma* spp.; *Eh*: *Ehrlichia* spp.; *Bb*: *Borrelia burgdorferi*; *Di*: *Dirofilaria immitis*; *Rc*: *Rickettsia conorii*; *Bc*: *Babesia canis*.

**Table 4:** Results of the binomial logistic regression evaluating statistical associations between potential risk factors and positivity to at least one pathogen in the dogs of the present study.

Factor	*p*	OR	95% CI
Male sex	0.831	1.045	0.700–1.558
7 or more years	0.005 *	1.903	1.215–2.981
At least one clinical sign	0.028 *	4.082	1.168–14.262
Two or more clinical signs	0.711	0.757	0.173–3.305
Prevention ectoparasites	0.497	1.165	0.750–1.811

* significant result; *p* = *p*-value, OR = odds ratio; CI = confidence interval.

## Data Availability

The original contributions presented in this study are included in the article. Further inquiries can be directed to the corresponding author.

## Abbreviations

The following abbreviations are used in this manuscript:

VBD	Vector-borne diseases
VBP	Vector-borne pathogens
